# Multiple strategies to improve sensitivity, speed and robustness of isothermal nucleic acid amplification for rapid pathogen detection

**DOI:** 10.1186/1472-6750-11-50

**Published:** 2011-05-11

**Authors:** Yanhong Tong, Bertrand Lemieux, Huimin Kong

**Affiliations:** 1 BioHelix Corp., Beverly, MA, USA

## Abstract

**Background:**

In the past decades the rapid growth of molecular diagnostics (based on either traditional PCR or isothermal amplification technologies) meet the demand for fast and accurate testing. Although isothermal amplification technologies have the advantages of low cost requirements for instruments, the further improvement on sensitivity, speed and robustness is a prerequisite for the applications in rapid pathogen detection, especially at point-of-care diagnostics. Here, we describe and explore several strategies to improve one of the isothermal technologies, helicase-dependent amplification (HDA).

**Results:**

Multiple strategies were approached to improve the overall performance of the isothermal amplification: the restriction endonuclease-mediated DNA helicase homing, macromolecular crowding agents, and the optimization of reaction enzyme mix. The effect of combing all strategies was compared with that of the individual strategy. With all of above methods, we are able to detect 50 copies of *Neisseria gonorrhoeae *DNA in just 20 minutes of amplification using a nearly instrument-free detection platform (BESt™ cassette).

**Conclusions:**

The strategies addressed in this proof-of-concept study are independent of expensive equipments, and are not limited to particular primers, targets or detection format. However, they make a large difference in assay performance. Some of them can be adjusted and applied to other formats of nucleic acid amplification. Furthermore, the strategies to improve the *in vitro *assays by maximally simulating the nature conditions may be useful in the general field of developing molecular assays. A new fast molecular assay for *Neisseria gonorrhoeae *has also been developed which has great potential to be used at point-of-care diagnostics.

## Background

Several platforms of isothermal nucleic acid amplification have been invented and developed in the past 18 years. However, so far most of them, such as transcription-mediated amplification (TMA) [1], rolling cycle amplification (RCA) [2], loop-mediated isothermal amplification (LAMP) [3], nucleic acid sequence based amplification (NASBA) [4], strand displacement amplification (SDA) [5], the helicase-dependent amplification (HDA) [6,7] require 1 hour or more of amplification time to detect <100 copies of template DNA. The improvement on speed (e.g. 30 minutes or less of amplification), sensitivity and robustness is not only important to the technologies themselves, but also a prerequisite for their applications in the field of point-of-care molecular diagnostics. Although accelerating the traditional nucleic acid amplification technology, like the polymerase chain reaction (PCR), is relatively straightforward from an engineering standpoint, all of the solutions proposed thus far (e.g., ABI 7500 system, Handylab chip-based Jaguar system) dramatically increase the cost of the technology, making it unattractive for point-of-care (POC) or decentralized laboratory diagnostics. These solutions are not suitable for isothermal amplification technologies because they focus on accelerating temperature shifts.

The focus of this study is to devise strategies to improve the speed, sensitivity and robustness of HDA reactions with <100 copies of input template, without relying on expensive instruments. Compared to PCR technology, HDA uses a helicase enzyme rather than heat to separate double-stranded nucleic acids. Like PCR, the simple reaction scheme requires a pair of primers, a protein mixture (helicase, single-stranded DNA binding protein and DNA polymerase), and buffer [6]. In our previous research, we have successfully developed real-time HDA assays that employ non-specific DNA intercalating dye (EvaGreen^®^), or sequence-specific fluorescent probes (TaqMan probes, MGB Eclipse probes) [8]. The specific amplification products can also be detected at end-point by lateral-flow strips in a handheld device called the BESt™cassette [9]. In this platform, asymmetric HDA is performed with biotin-labeled excess primer. The detection probe, complementary to the DNA strand extended from biotin-labeled primer, is labeled with either fluorescein (for the detection of target amplification) or digoxgenin (for the detection of internal control amplification). Since HDA assay mimics a process that occurs in nature (replicating DNA by using helicase to unwind DNA duplexes at a constant temperature), the best strategies to improve HDA also come from nature. Two strategies that maximally simulate the nature process are discussed here: restriction endonuclease-mediated DNA helicase homing that mimics the natural process of mismatch repair pathway, and macromolecular crowding agents that mimic the natural enzyme working environment. Further optimization of reaction enzyme mix is also discussed here. In addition, the improvement effects on speed, sensitivity and robustness are further explored by combining of aforementioned strategies. *Neisseria gonorrhoeae *(NG) was selected as a test case to demonstrate these innovations because it is the second most frequently reported sexually transmitted diseases in the United States, and there is a medical need for a rapid POC tests with greater sensitivity to detect this pathogen.

## Results and discussion

### Effects of restriction endonucleases

HDA uses helicase(s) to separate a DNA duplex. Helicase is not a sequence specific protein, thus helicase does not specifically recognize the target region. Therefore, whether helicase can efficiently separate the double-stranded nucleic acid, especially in the target region specified by forward and reverse primers, is an important rate-limiting factor or a factor highly related to the reaction robustness and sensitivity at low copy number of target detection.

**Figure 1:**
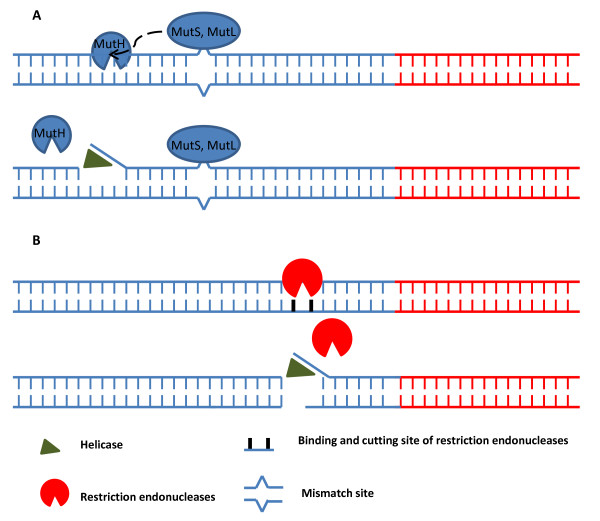
**Schematic model of restriction endonucleases effects on HDA**. Panel A.) Mechanism *in vivo*: The mismatch is recognized by MutS. MutS and MutL form complex to stimulate MutH which generates nick site of the DNA duplex near the mismatch position. UvrD (helicase) is then loaded, unwinds the DNA duplex at the nick, and extends toward the mismatch. Panel B.) Mechanism *in vitro*: Restriction endonucleases specifically cut the DNA duplex near the target sequence, generate blunt or 5' ss or 3' ss ends or nick site (if using nicking enzyme). UvrD is then loaded and unwinds the DNA duplex. Red lines represent target DNA sequence. Blue lines represent non-target DNA sequence.

The helicase used in thermophilic HDA [7] belongs to the mismatch repair system *in vivo*. Figure 1A shows the mechanism of *E. coli *UvrD mismatch repair system, which is the most understood system [10], and most likely resembles the mechanism through which thermophilic helicase operates. The *E. coli *system requires multiple accessory proteins (e.g. at least mutS, mutL and mutH) to generate nicks near the mismatch sites and then load the UvrD, which has high affinity in binding the ends of nucleic acid molecules [7,11]. However, from a manufacturing perspective, purifying so many accessory proteins to perform *in vitro *amplifications would be too costly. Although the nucleic acid substrates input into *in vitro *amplification reactions are sheared or nicked during the extraction and purification steps, the DNA ends generated by this process are randomly distributed along the nucleic acid substrates and are not target specific and thus not to aid HDA. As shown in Table 1 the Tt (Threshold time, is defined as the number of detection cycles required for the fluorescent signal to cross the threshold. It is a similar definition as cycle threshold for real-time PCR. The only difference is that the cycle in real-time PCR is thermal cycle. In order to compare the assay speed and robustness, the Tt value is converted to minutes by 1 Tt = 2 minutes. The set-up details of detection cycle are described in the Methods section) values of HDA amplification on low copy number targets are distributed widely over 3-6 minutes. We hypothesize that reactions with low copy number of targets benefit more obviously from a proximal nick or double strand break in the template nucleic acid than those with high copy number of targets. Because loading helicases near the rare amount of target region is a random event with low chance.

**Table 1:** Effects of restriction endonucleases (50 copies/assay).

	Tt (Min)	Min Tt (Min)	Max Tt (Min)	Max Difference of Tt (Min)
With MboI	26.88 ± 0.88	25.20	28.00	2.80
Without MboI	32.30 ± 2.70	29.02	37.84	8.82

We tested our hypothesis that double strand breaks can improve the loading efficiency of the helicase near a target sequence by evaluating the impact of restriction endonuclease digestion during HDA amplification. As shown in Figure 1B, specific restriction enzyme can be selected to cleave a specific sequence near the target sequence, generate 5'ss (single-stranded) ends, 3'ss ends or blunt ends to help recruiting and loading the helicase. Therefore, one simple protein for mediation of helicase homing can replace the functions of multiple accessory proteins *in vivo*. Indeed, previous studies had already used restriction enzymes to produce substrates for helicase unwinding assay *in vitro *[12,13]. So far more than 3000 restriction endonucleases with over two hundred different specificities have been isolated from bacteria [14]. This broad collection makes finding an enzyme which can cut the DNA duplex close to the target very straightforward. The "time-saver qualified enzymes" from New England BioLabs Inc. (Ipswich, MA) are ideal choices because these enzymes will digest about 1 μg of nucleic acid in about 5 minutes using about 1 μL of enzyme under recommended conditions. The fast speed of these enzymes makes it possible to add a restriction enzyme together with the HDA enzyme mix during the reaction set-up stage. And the limited set-up time (generally 2 to 5 minutes depending on the number of assays) will be enough for the selected restriction enzyme to generate sufficient numbers of DNA ends near the target sequence to accelerate the HDA reaction.

Ideal enzymes for restriction endonuclease based helicase homing: 1) specifically cut the site close to the target sequence (for example, less than 200 bp distance), but not the target sequence and internal control sequence; 2) belong to the group of "time-saver qualified enzymes"; 3) have incubation buffers that are compatible with the HDA reaction buffer; 4) have optimal enzyme working temperatures of 37°C or below [15]. In the case of the NG *PorA *target sequence, MboI can cut the DNA 100 bp upstream of the forward primer. To demonstrate this effect, we performed 9 HDA reactions (8 with 50 copies of starting template/reaction, 1 for NTC) with MboI (5 units/assay) and another 9 reactions without MboI side-by-side for comparison. As shown in Table 1 with the addition of MboI, the standard deviation of the Tt values reduced to less than 1 minute, and the maximum difference among 8 reactions was less than 3 minutes. Therefore, low copy number of target could be more consistently amplified. The average Tt was around 6 minutes faster than the corresponding assays performed without MboI. In addition, the robustness of the HDA reaction is greater with restriction enzyme than that without especially when the internal control sequence is included in the reaction. Because in some reactions performed without MboI, the internal control sequence is preferentially amplified instead of the low copy number of target (e.g. less than 50 copies/assay, data not shown here), and the sensitivity of the assay for the target sequence is reduced. Therefore, the speed, sensitivity and robustness are dramatically improved with the addition of restriction endonucleases.

### Effects of macromolecular crowding

*In vivo *biological reactions take place in a crowded environment in which around 20 to 40% of the total volume in the cell consists of macromolecules. Macromolecular crowding agents, such as Ficoll and Dextran, are known to increase the reaction speed of several enzymes, including DNA polymerase, RNA polymerase, ligase, endonucleases, exonucleases [16-20]. These agents can also affect DNA structure and stability [21]. We hypothesized that macromolecular crowding agents should enhance the efficiency of the HDA reaction.

**Figure 2:**
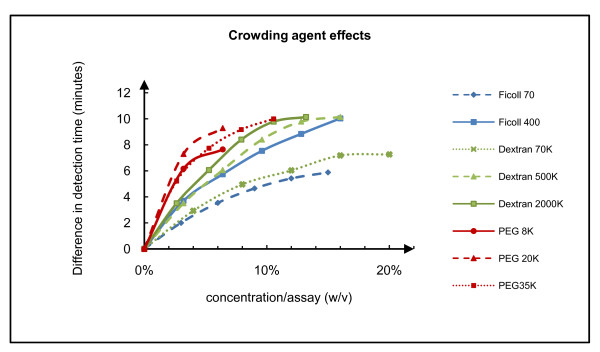
**Effects of macromolecular crowding reagents**. The effects are shown as the difference in detection time (calculated by: 2 × (Tt_reaction without crowding agent_- Tt_reaction with crowding agent _)) relative to non chemical control reactions versus crowding agents concentration in the final assay. The blue groups represent the effects of Ficoll, the green groups represent the effects of Dextran, the red groups represent the effects of PEG. The maximal tested concentration of each chemical is limited by its solubility (stock concentration) or viscosity (transferable or not by pipette).

In order to examine the effect of macromolecular crowding on HDA, we tested the effect of numerous agents; *i.e*., polyethylene glycol (PEG) of 8 kilodaltons (8 K), PEG 20 K, PEG 35 K, Ficoll 70, Ficoll 400, Dextran 70 K, Dextran 500 K, Dextran 2000 K. The Tt values of amplifications with 50,000 copies of NG genomic DNA performed with different concentrations of these crowding agents were compared to the control reactions performed without the latter. As shown in Figure 2, HDA reactions performed under the conditions of macromolecular crowding were faster than the control reactions and the higher molecular weight crowding agents had the greatest effects on reaction speed. However, higher molecular weight chemicals also increased the viscosity of the solution, such that pipetting accuracy and mixing efficiency were affected. The upper limit of concentration of each chemical in HDA reaction was also limited by its solubility in stock solution. Although the PEG group had the most obvious effects, it also increased the incidence of primer dimer formation as measured by melt curve analysis (data not shown). Therefore, Ficoll and Dextran in final concentrations ranging from 5% to15% are better crowding agents for accelerating HDA.

### Improvement of speed, sensitivity and robustness by combination of strategies

The speed and robustness of the HDA system is also highly dependent on the synchronization of three enzymes: helicase, single-stranded DNA binding protein and large fragment of Bst DNA polymerase. By increasing the concentration of the constituent enzymes, while maintaining the relative proportions of each type of enzyme constant, we were able to evaluate the speed and accuracy of the HDA reaction. Enzyme concentrations ranging from one-fold (1X) to four-fold (4X) were found to improve the overall reaction speed. Increasing primer concentration also improves speed but can result in a loss in accuracy evidenced by primer dimer formation. Table 2 summarizes the improvement in speed by increasing enzyme mix concentration from 1× to 3×.

**Table 2:** Comparison of speed improvements from individual strategy vs. combination of all strategies (50 copies/assay).

Condition	3× IsoAmp III	MboI	10% Ficoll	Average Tt (Min)
1 (control)	-	-	-	33.40 ± 1.88
2	√	-	-	20.51 ± 0.78
3	-	-	√	24.43 ± 1.79
4	√	√	√	14.81 ± 0.51

"-" represents absence of the condition.

"√"represents existence of the condition.

In order to maximally optimize the assay for rapid and robust detection of pathogen within 30 minutes, one or more aforementioned strategies were implemented and compared. Table 2 summarizes the outcome of 4 experiments where combination of the three strategies is compared side-by-side with single strategy, as well as without any of above strategies (tested with 50 copies of NG genomic DNA per reaction, only the examples of 3× enzyme mix are listed here). Greatest speed, robustness and sensitivity are achieved when all strategies are combined.

**Figure 3:**
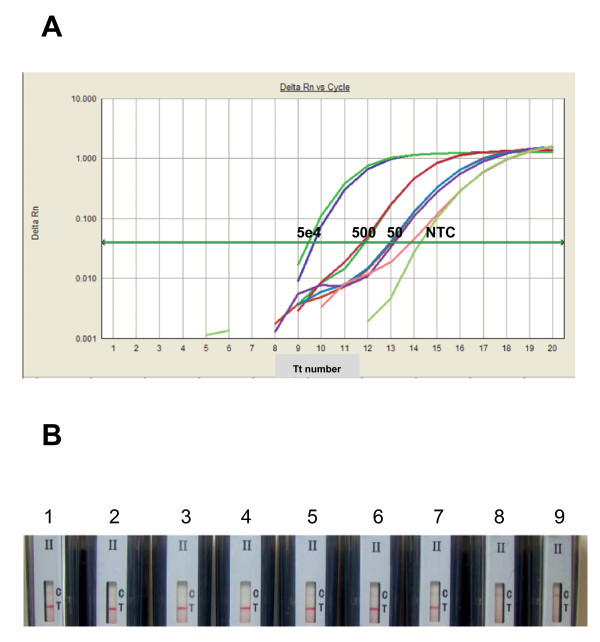
**Rapid amplification and detection of *Neisseria gonorrhea *genomic DNA**. Panel A) Fluorescence monitoring in real-time is shown as semi-log plot of fluorescence intensity versus Tt number where each cycle is 1 minute-long. The copy numbers of input template are labeled on the figure. Panel B) Type II BESt™ cassette detection of 5e4 copies/assay (strips 1-2), 500 copies/assay (strips 3-4), 50 copies/assay (strips 5-7), and non template control (NTC) (strips 8-9). The signal from NTC (either in panel A or B) is the amplification signal from the internal control sequence. The IC sequence shares the same primer sequence with the detected target gene of *Neisseria gonorrhoeae*, just with different internal nucleic acid sequence between the primers. The IC sequence is cloned into a plasmid. When a reaction is set-up, the IC plasmid is premixed with all of the other reaction components (except the target DNA or tested clinical samples) to prepare the master mix of the assay. When there is no target template of *Neisseria gonorrhoeae *(or negative clinical samples) in the assay, the IC sequence is amplified. Because the different internal sequence between the IC sequence and the target sequence of *Neisseria gonorrhoeae*, different probes are designed and included in the reaction. This can be differentiated from T line (Test line: for the detection of target DNA amplification products by target probe NGP) and C line (Control line: for the detection of internal control amplification products by IC probe NGICP) in Panel B.

In order to test the optimal performance of the assay and the feasibility for type II BESt™ cassette detection after 20-minutes amplification, the reaction was set up with the final conditions as: standard HDA condition with addition of 4× IsoAmp III, 10 μL of 40% Ficoll 400, 5 units of MboI and 1e5 copies of internal control (IC) DNA. The purpose of including internal control DNA in each clinical assay is to monitor the potential inhibitors from clinical specimens which might generate false negative results. Generally, one kind of detection platform is used for each assay (either real-time based detection or BESt™ cassette based detection). However, in order to demonstrate that the improved Tt value (smaller number) also indicates that the shortened amplification time required for end-point detection, two detection methods were used at the same time in this study. Real-time detection dye (EvaGreen^® ^and ROX) was included in the assay to evaluate the speed by Tt values, and the probes for cassette detection were also included in the reaction to verify the detection performance by cassette (as described in the Methods section). Assays were incubated in the ABI 7300 with the modified program to monitor the improved speed: 20 cycles of 66°C for 5 seconds, and 65°C for 55 seconds with data collection and fluorescence signal being collected at the end of each cycle (1 Tt number = 1 minute). After 20 minutes (20 cycles), the reaction tubes were immediately placed into type II BESt™ cassettes for detection of reaction products according to the package insert supplied by the vendor [9]. The results are shown in Figure 3. The Tt was less than 15 minutes for both 50 copies of target and NTC (where the internal control sequence was amplified in the absence of target) (Figure 3A). And the positive test lines were shown on the strips from the 50000, 500 and 50 copies of NG genomic DNA input. The control lines were shown on the strips from the non NG template control, where the amplified internal control sequence was detected here (Figure 3B). The data also demonstrated that the improvements of speed and robustness are not depended on particular detection format.

The improvement on sensitivity, speed, and robustness is just a prerequisite for applying HDA technology to rapid pathogen detection at the POC. However, since clinical samples always have inhibitors, sample preparation is generally required for molecular diagnostic assays. Simple and fast sample preparation for the detection of *Neisseria gonorrhoeae *from urine has been developed for the feasibility study: 0.5 mL of urine was added to each 2 mL urine collection tube, and spun down for 5 minutes with highest speed at benchtop centrifuge (>10 k rpm). The supernatant was decanted, and 200 μL of sample dilution buffer was added to the collection tube. The closed collection tube was heated at 95°C for 5 minutes and then spun down again for 5 minutes at the same conditions as the first spin. 5 μL of sample was subjected to each HDA assay. By this method, most of inhibitors can be removed and diluted. Preliminary clinical study was performed with pooled negative frozen urine samples and additional 58 clinical specimens (5 positive and 53 negative samples, estimated by Abbott CT/GC kit). The result is summarized in Table 3. 5 of the negative samples showed inhibitions, but it could be resolved by further dilution (e.g. 2-8 fold dilution) and/or longer amplification time (30 minutes). However, the final format of the assay (including sample preparation) will be determined and validated by further study with fresh urine samples in the next phase.

**Table 3:** Preliminary study with clinical urine samples (compared with Abbott CT/GC kit).

		Reference Method (Abbott CT/GC kit)		
		
		Positive	Negative	Total
**HDA Assay**	**Positive**	5	0	5
	**Negative**	0	53	53
	
	**Total**	5	53	58

## Conclusion

In summary, we have explored several strategies to improve the HDA performance in terms of speed, sensitivity and robustness. The strategies are independent of expensive equipments, and are not limited to particular primers or targets. Some of them can be adjusted and applied to other formats of nucleic acid amplification. Furthermore, the strategies to improve the *in vitro *assays by maximally simulating the nature conditions may be applied in the general field of developing molecular assays. By these strategies, HDA amplification can be shortened to around 20 minutes. Therefore, it is feasible to apply HDA for rapid and sensitive pathogen detection at point-of-care diagnostics.

## Methods

### Materials

Quantitated *Neisseria gonorrhoeae *genomic DNA of known concentration (in the range of 1-2 × 10^4 ^copies/μL) was purchased from Advanced Biotechnologies Inc. (Columbia, MD). In order to keep the assay consistent and comparable, and avoid the effects of freeze-thaw, the DNA was aliquoted for single use and stored at the recommended conditions based on the information from the package insert. All the oligonucleotides were from either IDT (Coralville, IA) or Eurogentec (Fremont, CA). All of the chemicals (Ficoll, Dextran, PEG) used in this study were from Sigma-Aldrich (St. Louis, MO). Restriction endonucleases were purchased from New England Biolabs (Ipswich, MA). Type II BESt™ cassette, IsoAmp III enzyme mix and all the other HDA reagents were from BioHelix Corp. (Beverly, MA).

### Standard HDA assay for *Neisseria gonorrhoeae*

*PorA *was selected as the target gene of NG [22]. The primers, probes, and internal control plasmid were designed and optimized as described by Chow et al. [9]. The primers/probes sequences were: primer BioNGF: Biotin-CCGGTTTCAGCGGCAGCATTCAATTT; primer NGR: TTTCCAGCGTGAAAGTAGCAGGCGTA; probe for NG (NGP, labeled with fluorescein): CTGTTTTGACTCGG/36-FAM/; probe for internal control (NGICP, labeled with digoxgenin): GTGCGGACTCTTTG/3Dig_N/. The modifications linked to the primers or probes are required for specific product detection by Type II BESt™ cassette at the end-point of isothermal amplification. For each 50 μL real-time HDA assay, the reactions were set up by mixing: 150 nM of BioNGF, 30 nM of NGR, 30 nM of NGP, 50 nM of NGICP, 1XABII buffer, 4.2 mM MgSO_4_, 35 mM NaCl, 3.5 μL dNTP-solution, 0.5 μL EvaGreen^® ^(Biotium, Hayward, CA), 1 μL ROX (Invitrogen, Carlsbad, CA), 1× IsoAmp III enzyme mix and different amount of template. Reactions were incubated in the ABI 7300 with the following program: 30 cycles of 66°C for 5 seconds, and 65°C for 115 seconds with data collection and fluorescence signal being collected at the end of each cycle (Note: the cycling of temperature is not required for the isothermal HDA assay, however, it is required by ABI 7300 for data collection. It is the cycle of fluorescent signal detection. If the assay is performed in a LightCycler 2.0, the program can be set up as 30 cycles of 65°C for 2 minutes with data collection at the end of each cycle), followed by a melt curve analysis. Therefore, 1 cycle number equals 2 minutes. When the products were analyzed by Type II BESt™ cassette, the melt curve analysis were not performed, instead the reaction tubes were inserted to the cassette for specific detection of target and/or internal control amplification.

## Abbreviations

HDA: helicase-dependent amplification; NG: *Neisseria gonorrhoeae*; PEG: polyethylene glycol; POC: point-of-care; IC: internal control; NTC: non template control.

## Authors' contributions

YT carried out the studies, and drafted the manuscript. BL and HK supervised the overall research, revised and finalized the manuscript. All authors read and approved the final manuscript.
